# Supplementary material for: Availability, affordability and access to essential medications for asthma and chronic obstructive pulmonary disease in three low- and middle-income country settings
Source: PLOS Glob Public Health. 2022 Dec 16;2(12):e0001309. doi: 10.1371/journal.pgph.0001309 (PMC10021856; doi:10.1371/journal.pgph.0001309)
Supplement: S1 Data — (DOCX) [file pgph.0001309.s001.docx]

**S1 Data**

**Nepal**

Nepal is a low-income country located in Southeast Asia with a total population of 28.6 million, of which 82% is rural.^1^ Nepal’s GDP is $30 billion, with 25% of the population living below the national poverty line. The current ratio of physicians per person are 1:1,742. The minimum wage at the time of data collection was Rs 13,450 per month ($120). Bhaktapur is an urban population center located eight miles east of Kathmandu. The majority of the estimated 80,000 inhabitants of Bhaktapur municipality are either craftsman or businessmen, while many migrants come to work in the outskirts at brick or carpet factories. Although Nepal has a national health insurance program, there is poor enrollment (11%) and high dropout.^2^ Medications are provided free of charge at public facilities when available. Out of pocket spending accounts for 55% of health care spending in Nepal.^3^

**Peru**

Peru is an upper-middle income country located in South America with a population of 30.5 million, 10 million of whom live in the capital (Lima), and 78.1% of whom live in urban areas.^1^ Peru’s GDP is 226 billion, and 26% of the population lives below the national poverty line. The current ratio of physicians per person are 1:1,116. In 2017, the minimum wage in Peru is 850 soles per month ($206). Pampas de San Juan de Miraflores and Villa El Salvador, where the surveys took place are urban communities in southern Lima. Peru has a decentralized healthcare system, the majority (74%) of the population receiving health insurance through the Peruvian Ministry of Health of the Social Security (EsSalud). Insured individuals can receive generic versions of certain respiratory medications, such as salbutamol and budesonide, at no cost, provided that the medications are available at public pharmacies. Private pharmacies generally do not require a prescription, but medication costs are covered by insurance only in rare cases.

**Uganda**

Uganda is a low-income country located in East Africa with a total population of 44 million, and a large rural population (75.6%).^1^ Uganda’s GDP is $35 billion with 19.5% living below the national poverty line. Nakaseke is a rural district in central Uganda.^4^ Most of the inhabitants (75%) are subsistence farmers and over 60% of them live on less than 45,000 Ugandan Shillings ($12) per month. The ratio of physicians/nurses per person are 1:25,000 and 1:5,000, respectively making Nakaseke one of the most under resourced health districts in Uganda. The lowest minimum wage in a public sector government job at the time of survey was 187,660 Ugandan Shillings per month ($51) for an entry point support staff worker in the public service division.^4^ Currently, only 1-2% of the population is privately insured. Limited health care services are available free of charge at government referral hospitals. As a result, out of pocket costs are very high for health expenses. A new health insurance scheme is planning to be rolled out that will subsidize the cost of healthcare.

**References**

1. Total Population. World Bank <http://data.worldbank.org/indicator/SP.POP.TOTL>. Accessed August 19^th^ 2022

2. Ranabhat CL, Subedi R, Karn S. Status and determinants of enrollment and dropout of health insurance in Nepal: an explorative study. Cost Effectiveness and Resource Allocation. 2020;18(1):1-13.

3. Organization WH. Prevention and control of noncommunicable diseases: guidelines for primary health care in low resource settings: World Health Organization; 2012.

4. Robertson NM, Nagourney EM, Pollard SL, Siddharthan T, Kalyesubula R, Surkan PJ, Hurst JR, Checkley W, Kirenga BJ. Urban-Rural Disparities in Chronic Obstructive Pulmonary Disease Management and Access in Uganda. Chronic Obstructive Pulmonary Diseases: Journal of the COPD Foundation. 2019;6(1):17.

5. Salary Structure for Political Leaders for Financial Services 2018/2019. Uganda Ministry of Public Service. Kampala, Uganda. 2019 https://www.publicservice.go.ug/media/resources/Salary%20Structure%20FY%20201819%20Schedule%201%20-%2012.pdf". Accessed August 19^th^ 2022
