# Supplementary material for: Availability, affordability and access to essential medications for asthma and chronic obstructive pulmonary disease in three low- and middle-income country settings
Source: PLOS Glob Public Health. 2022 Dec 16;2(12):e0001309. doi: 10.1371/journal.pgph.0001309 (PMC10021856; doi:10.1371/journal.pgph.0001309)
Supplement: Attachment — Submitted filename: Reviewer Comments 9.8.docx [file pgph.0001309.s002.docx]

To the Editors of PLoS Global Public Health,

We appreciate the thoughtful and thorough review of the manuscript “Availability, Affordability and Access to Essential Medications for Asthma and Chronic Obstructive Pulmonary Disease in Three Low- and Middle-Income Country Settings”. We believe that the following edits will substantially improve the manuscript.

Sincerely,

Trishul Siddharthan

Reviewer #1: Reviewer’s comments

This study by Siddharthan T et al is a well-written study highlighting an important area of inequity in access to essential medicines for asthma and COPD in three low-and middle-income countries. It comprehensively assesses the availability, pricing, and affordability of essential medicines as recommended by the WHO essential medicines list. Despite its strengths, there are areas that need to be revised in the manuscript to improve its quality.

Thank you

Major revisions.
1. Information on the availability of LAMA and LABA combination, a key medicine in the management of COPD, is missing in the abstract. I would advise that this information is added.

We have added this information to the abstract.

2. There is little reference to studies evaluating the availability and affordability of asthma and COPD medicines performed in similar LMIC settings in the discussion section. The authors should include some of these key studies below to improve their discussion section:
- Kibirige D et al. Availability and affordability of medicines and diagnostic tests recommended for management of asthma and chronic obstructive pulmonary disease in sub-Saharan Africa: a systematic review. Allergy Asthma Clin Immunol. 2019 Mar 7;15:14.
- Mendis S, Fukino K, Cameron A, Laing R, Filipe A Jr, Khatib O, Leowski J, Ewen M. The availability and affordability of selected essential medicines for chronic diseases in six low- and middle-income countries. Bull World Health Organ. 2007;85:279–88.
- Cameron A, Ewen M, Ross-Degnan D, Ball D, Laing R. Medicine prices, availability, and affordability in 36 developing and middle-income countries: a secondary analysis. Lancet. 2009;373:240–9.
- Mendis S, Al Bashir I, Dissanayake L, Varghese C, Fadhil I, Marhe E, Sambo B, Mehta F, Elsayad H, Sow I, et al. Gaps in capacity in primary care in low-resource settings for implementation of essential noncommunicable disease interventions. Int J Hypertens. 2012;2012:584041.
- Ozoh OB et al. Asthma and COPD medicines availability and affordability in Nigeria. Trop Med Int Health. 2021 Jan;26(1):54-65.

Thank you for the references. We have modified our discussion section and cited these studies accordingly.

3. The authors should include information about the wages for the lowest paid unskilled government worker that was used to assess the affordability of these medicines in each country/study setting.

This information has been included in the supplementary material.

4. In relation to my above comment, I find the reported affordability of salbutamol inhaler as treatment of intermittent asthma very inaccurate.
The authors should refer to the Ministry of Public Service, Republic of Uganda website and this link below for guidance on the wage structure in 2019/2020, a period when the study was conducted: <https://www.publicservice.go.ug/media/resources/Salary%20Structure%20FY%20201819%20Schedule%201%20-%2012.pdf>
According to the Public Service wage structure in Uganda, the lowest-paid unskilled government worker is at the U8 level. Using that information, please revise your results on the affordability of the drug in Uganda.

The wage that was used in this analysis comes from the same source: M8 187,660 per month, as referenced in the supplement.

5. There is a study limitation of lack of information on the availability and affordability of leukotriene antagonists which are key medicines in the management of asthma.

We have included this as a limitation.

Minor comments
The manuscript has several typographical errors that need extensive revision.
An example of these errors is shown below:
- On the 2nd last line of page 8, the number of health facilities where Salmeterol was present is missing (30.3%, n=……….).
- On page 9, Formoterol is misspelled (section on availability of long-acting antimuscarinic agents).
- On page 10, the author’s name is misspelt (Kibirige NOT Kabirige).

We apologize. These errors have been corrected.

- Affordability should be corrected and expressed as days’ wages throughout the manuscript (see the section on the affordability of essential medicines and table 2).

We have modified the manuscript to express affordability in day’s wages.

Reviewer #2: General
While I agree that such availability studies are very useful for a given country, I am less convinced that such a cross-country study has much additional benefit. Also, for this study, the question comes up: so what? It seems that the only conclusion is that the availability is usually higher in private pharmacies (with a few exceptions) and that prices vary between the three countries; and that the range of medicines in Nepal seems wider. So what can we do with this information?

I would recommend that you try to expand the discussion a little further, and formulate hypotheses to explain the large price differences between the three countries. It could also be discussed why there are so many combination medicines in Nepal – I am rather sure that very few of such combinations are on the WHO Model List. It would also be very interesting if you formulated some practical advice to each of the three countries about how to improve their particular situation.

Thank you for this suggestion. We agree that there needs to be further clarification as to the added value of the present study. We have added a section in the introduction, as well as have added to the discussion regarding medication availability in Nepal.

Methods
What sort of region were these – urban, semi-urban, rural? Please give some additional information
Sample selection: I am not very sure about how the medicines included in the survey were selected – especially in view of the many non-essential combinations used in Nepal. Did you measure and report any drug in the selected categories that you could find in the private pharmacies surveyed? Why did you not limit yourself to medicines on the WHO Model List? I would not like the paper to suggest that the many non-essential medicines observed as available in Nepal should actually have been available in the other two countries. At least, in the discussion I recommend that you reflect on the differences between the WHO Model List and the medicines actually found in the pharmacies in the three countries.

We have included detailed information of each site in the supplement. To ascertain availability, pharmacists were provided a list of all available respiratory medications on national formularies and asked whether they had any in stock. Additionally, pharmacists were asked whether they had any medications available (or combinations) not on the formulary. The WHO essential medication list is not comprehensive (as in the case of Nepal), and the wide variety informs actual patient experience at each site studied. We agree with the statement that the experience in Nepal should not be replicated and represents a barrier to effective care (particularly given combination antibiotic therapies). The sites were chosen based on a population-based study related to COPD diagnosis and management (Global Excellence in COPD Outcomes)

Data analysis
Line 12: Check grammar
Iine 14: GOLD and GINA first time in full

Thank you, these errors have been corrected.

Results: There is no need to describe all figures and percentages again as text as they read better as tables. So I suggest to remove all the “drug – specific” text subsections and restrict yourself to referring to the two tables, and (perhaps) mentioning one or two specific important facts.

Results: Think the number of public pharmacies studied is so small (both absolutely as relatively) that I doubt that you can make a meaningful comparison between the public and private sector. Can you please check your statistics whether any differences observed are statistically signifiant?

Thank you for this suggestion. We have modified the manuscript to include a comparison of differences in public vs private drug availability, as well as differences in affordability between country settings.

Reviewer #3: This study is to provide information on availability, affordability, and access to essential medicines for selected respiratory diseases in three countries. Below are a few major concerns:
1. Introduction: Although the manuscript highlighted the scale of respiratory disease, there is little information on (1) reasons why it is important to study availability, affordability, and access in the three countries. In another word, what is the uniqueness of this study; (2) what has been done previously in the three countries; and (3) what gaps would this study fill in?

2. Methods: The methodology to carry out the study and analysis is not adequately described. The method sector also needs to be restructured, including study design, sampling, measurement, data collection and analysis.

We have restructured the methods section to include the prespecified headings.

2.1. The selection of countries and catchment areas is not described in detail, particularly the selection of the catchment areas. how were they selected? Were they selected through random sampling? were they representative of the country's catchment areas?

These details are included in the supplement, given space limitations in the primary manuscript.

2.2. There is no description of the drug system in the three countries to prepare readers to understand the study setting, such as the pharmaceutical market share between the public and private sectors.

We have added this information to the supplementary materials.

2.3. The measurements should be described in more detail under a separate heading. The manuscript should be clear about how availability, affordability, and access were measured. The measure should not be under the data analysis. The development of indicators should also be described in more detail. Key parameters should be provided, such as the daily wage for each country.

We have restructured this section as outlined and apologize for not including the supplement in original version.

2.4. Most statistical analysis is descriptive. Further analysis could be explored, such as the comparison of availability and affordability among sites and drugs, and between sectors.

We have modified the manuscript to include an analysis comparing availability and affordability across sites.

3. Results
3.1. suggest conducting further analysis to provide comparative analysis.
3.2. Table 2. The days to pay for treatment for Uganda are extremely high. What is the daily wage used for comparisons? According to <https://wageindicator.org/salary/minimum-wage/uganda>, the minimum wage per month is UGX 130,000, which is equivalent to $34.38 per month or 1.15 per day. The mean total days for Uganda would be about 3 days, rather than 54 days.

The minimum wage in 2019 for a government worker was 187,660 UGX. We apologize for the mistake, which has been corrected in the table as well as through out manuscript.

4. discussion
4.1. The discussion should focus more on explaining why there is a shortage in some countries for particular medicines and how it is related to the drug delivery systems.

We have added this information into the discussion including heterogeneity in medication availability at study sites.
